# Comparing a Multimedia Digital Informed Consent Tool With Traditional Paper-Based Methods: Randomized Controlled Trial

**DOI:** 10.2196/20458

**Published:** 2021-10-19

**Authors:** Fuad Abujarad, Peter Peduzzi, Sophia Mun, Kristina Carlson, Chelsea Edwards, James Dziura, Cynthia Brandt, Sandra Alfano, Geoffrey Chupp

**Affiliations:** 1 Department of Emergency Medicine School of Medicine Yale University New Haven, CT United States; 2 Department of Biostatistics School of Public Health Yale University New Haven, CT United States; 3 Department of Chronic Disease Epidemiology School of Public Health Yale University New Haven, CT United States; 4 VA Connecticut New Haven, CT United States; 5 Department of Internal Medicine School of Medicine Yale University New Haven, CT United States

**Keywords:** digital consent, digital health, e-consent, informed consent, mobile phone

## Abstract

**Background:**

The traditional informed consent (IC) process rarely emphasizes research participants’ comprehension of medical information, leaving them vulnerable to unknown risks and consequences associated with procedures or studies.

**Objective:**

This paper explores how we evaluated the feasibility of a digital health tool called *Virtual Multimedia Interactive Informed Consent* (VIC) for advancing the IC process and compared the results with traditional paper-based methods of IC.

**Methods:**

Using digital health and web-based coaching, we developed the VIC tool that uses multimedia and other digital features to improve the current IC process. The tool was developed on the basis of the user-centered design process and Mayer’s cognitive theory of multimedia learning. This study is a randomized controlled trial that compares the feasibility of VIC with standard paper consent to understand the impact of interactive digital consent. Participants were recruited from the Winchester Chest Clinic at Yale New Haven Hospital in New Haven, Connecticut, and healthy individuals were recruited from the community using fliers. In this coordinator-assisted trial, participants were randomized to complete the IC process using VIC on the iPad or with traditional paper consent. The study was conducted at the Winchester Chest Clinic, and the outcomes were self-assessed through coordinator-administered questionnaires.

**Results:**

A total of 50 participants were recruited in the study (VIC, n=25; paper, n=25). The participants in both groups had high comprehension. VIC participants reported higher satisfaction, higher perceived ease of use, higher ability to complete the consent independently, and shorter perceived time to complete the consent process.

**Conclusions:**

The use of dynamic, interactive audiovisual elements in VIC may improve participants’ satisfaction and facilitate the IC process. We believe that using VIC in an ongoing, real-world study rather than a hypothetical study improved the reliability of our findings, which demonstrates VIC’s potential to improve research participants’ comprehension and the overall process of IC.

**Trial Registration:**

ClinicalTrials.gov NCT02537886; https://clinicaltrials.gov/ct2/show/NCT02537886

## Introduction

### Background

Informed consent (IC) is essential for upholding ethical conduct in research and medical treatment. The goal of the IC process is to provide research participants with sufficient information about the proposed research so that the participants can make an autonomous decision regarding their health and well-being [1-4].

Despite the implications and importance of the IC process, the Joint Commission for Transforming Healthcare has reported that an estimated 60% to 70% of individuals do not read or understand the information contained in the consent form, and 44% of the participants signing the IC documents do not understand the nature of the proposed procedure [5]. Although many providers have opted for electronic IC in an attempt to mitigate these issues, this method usually results in a mere electronic version of the standard paper-based form and does not address participant comprehension [6]. This lack of sufficient information and participant comprehension in the IC process negatively affects participant safety [7].

### Related Works

Several studies have compared the efficacy of digital innovations in IC versus traditional paper consent. A systematic review conducted in 2019 to compare the 2 methods in published studies between 2012 and 2018 concluded that 67% of the included studies reported a positive effect on at least one of the studied outcomes. The efficacy of innovative interventions appeared high for interactive multimedia, with a positive effect on participants’ comprehension and no negative effect on satisfaction or participation [8].

Another study that compared different methods of providing the same information in different formats to participants found that presenting plain text to participants with only audio narration proved to be the least effective when compared with other methods such as animated video or comics [9].

Several digital platforms have been developed to improve the consent process, and although their findings have been shown to be more effective than paper IC, either in a real study or a hypothetical study, some of these systems lacked 1 or more of the features that make Virtual Multimedia Interactive Informed Consent (VIC) unique [10-16]. These features include the use of avatars, supporting other languages, multimedia support, text-to-speech, quizzes and surveys, teach-back technique, accessibility among blind and deaf people, and electronic signature features [17].

### Objective

Existing research suggests that the use of digital health interventions, such as virtual coaching and mobile apps, along with interactive audio and visual elements in a participant-centered IC, can increase the participant’s interest and retention [3,18-21]. We applied these principles in the design, development, refinement, and testing of our web-based digital IC tool, *VIC* [17], and compared the results of our feasibility study with those of traditional paper consent methods.

### VIC Tool

The initial concept of the VIC tool was developed on the basis of previous work, literature findings in IC research, participant input, and subject matter expert interviews [17]. The theoretical framework of VIC is based on Mayer’s cognitive theory of multimedia learning, and the principle that the use of multimedia in the presentation of the IC process will improve participant comprehension [22-25]. We adopted the user-centered design approach to design and develop a fully functional tool. Before using our tool, we conducted a usability study to evaluate user acceptability and satisfaction for the biorepository research study. Our tool uses virtual coaching with automated text-to-speech translation to conduct a brief and virtual interview with participants via tablet computers. VIC also features a comprehensive multimedia library (eg, video clips, animations, and presentations) to explain the risks, benefits, and alternatives of the proposed treatment or clinical study to enhance participants’ awareness [17].

**Figure 1:**
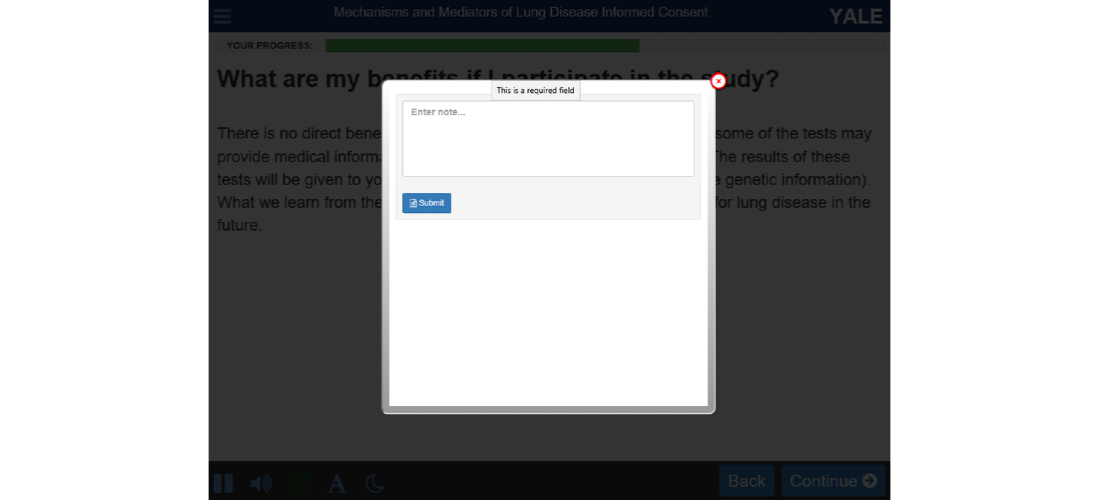
Virtual Multimedia Interactive Informed Consent features screenshot.

Our tool presents IC materials to participants with the option of going back and forth through each section as well as the ability to click on links within the sections, if desired, to drill down for more information. In addition, the VIC tool has an option to assess participant comprehension with automated quizzes, which can help emphasize the information presented. VIC provides many features and functions, including internet access to the consent, retrievable electronic records of IC, electronic signatures, and the potential for seamless integration with the electronic health record (Multimedia Appendix 1 and Figure 1). Moreover, VIC includes extensive security strategies that maintain the confidentiality and privacy of both participants and clinical information. It also provides access to the IC content via the internet before, during, and after the study or procedure, allowing the participants to benefit from supplemental resources as well [17].

Although more than half of the feasibility studies in the existing literature use hypothetical scenarios to test enhanced IC, we believe that the use of an actual research study improves the validity, accuracy, and reliability of the results [26,27]. This study is a randomized controlled trial to test VIC with participants involved in a real-world study and evaluated the tool’s feasibility and utility compared with the standard, paper-based IC.

## Methods

### Trial Design

This study was a randomized controlled trial to test the feasibility of VIC in an ongoing, real-world biorepository research study titled “Yale Center for Asthma and Airway Disease Mechanisms and Mediators of Chronic Lung Disease Study” (GenEx 2.0). GenEx 2.0, the parent study for VIC, evaluated the pathophysiology and heterogeneity of airway disease in participants using several procedures, including a coordinator-administered, self-assessed questionnaire, lung function testing, blood draw, and hypertonic saline–induced sputum induction at the Winchester Chest Clinic (WCC). A detailed IC document was required so that the participants understood not only the risks and benefits of the procedures but also the ramifications of contributing biological and genetic samples to the GenEx 2.0 study.

Using the parent study’s existing infrastructure and participants, the VIC trial recruited individuals and randomly assigned them to receive either the existing GenEx 2.0 paper consent document (control arm) or consent on the tablet through the VIC tool (intervention arm). Of the eligible participants, the allocation ratio for each group was 1:1. For both arms, the study coordinator responded to any questions from the participant regarding the IC process. After the consenting process, participants from both groups started the GenEx 2.0, which took an average of 2 hours to complete. The outcomes for the VIC trial were self-assessed using coordinator-administrated questionnaires at the WCC. Immediately following the GenEx 2.0, we surveyed the participants regarding their comprehension and satisfaction of the study to evaluate the effects of the VIC intervention.

### Participants

The GenEx 2.0 study recruited participants with lung disease from the WCC at Yale New Haven Hospital in New Haven (CT) and healthy individuals from the community using fliers. We approached the participants who were considered for the parent study and asked if they were interested in participating in the VIC trial in addition to their participation in GenEx 2.0. For VIC participants, the trial included web-based components in addition to face-to-face components for the survey, or was conducted primarily face-to-face depending on the arm to which the participant was assigned.

The participants were eligible for both GenEx 2.0 and the VIC trial if they (1) spoke English, (2) were older than 21 years, (3) provided an email address, and (4) were willing to use an iPad. Computer literacy was not required for eligibility. The participants were excluded from the GenEx 2.0 study for (1) having a smoking history of more than 10 packs a year, (2) being active smokers within the past year, or (3) having other chronic lung disease or asthma variants. In addition, GenEx 2.0 participants were excluded from participation in the VIC trial if they were (1) not able to safely undergo the studies required for participation, (2) were unable to read or understand English, (3) refused to participate, or (4) had participated in the GenEx 2.0 trial in the past.

No participants withdrew during the study period from the VIC trial.

### Randomization

Eligible and consenting participants were randomized to receive IC through standard paper consent or digital consent through the VIC tool. Because of the small sample size, we used the method of minimization [27] first to achieve balance on the following demographic characteristics—gender, race, education, employment type, marital status, household income, and technology confidence (Table 1). A computer algorithm belonging to the VIC back-end system maintained a record of all enrolled participants and automatically generated the randomization sequence after minimization. The study coordinator who handled enrollment would select a button in the VIC back-end system to prompt the randomization process, which would then display to which arm (control or intervention) the participant was randomized.

**Table 1:** Participant characteristics by method of informed consent administration (N=50).

Characteristic			VIC^a^ (n=25)		Paper (n=25)
Age (years), mean (SD)			47.1 (15.3)		37.7 (14.7)
**Gender, n (%)**					
	Male	9 (36)		10 (40)	
	Female	16 (64)		15 (60)	
**Race, n (%)**					
	White	17 (68)		15 (60)	
	Black or African American	6 (24)		6 (24)	
	Native American or American Indian	0 (0)		0 (0)	
	Asian or Pacific Islander	2 (8)		3 (12)	
	Other	0 (0)		1 (4)	
**Ethnicity, n (%)**					
	Hispanic or Latino	1 (4)		4 (16)	
	Non-Hispanic or Latino	24 (96)		21 (84)	
**Employment, n (%)**					
	Full-time	10 (40)		10 (40)	
	Part-time	3 (12)		5 (20)	
	Not employed	12 (48)		10 (40)	
**Education, n (%)**					
	High school graduate or GED^b^	7 (28)		6 (24)	
	Some college or associate’s degree	6 (24)		5 (20)	
	College degree (bachelor’s program)	5 (20)		7 (28)	
	Graduate or professional degree	7 (28)		6 (24)	
	Other	0 (0)		1 (4)	
**Household income before tax (US $), n (%)**					
	<50,000	15 (60)		19 (76)	
	50,000-99,999	4 (16)		4 (16)	
	≥100,000	6 (24)		2 (8)	
**Marital status, n (%)**					
	Single or widowed	14 (56)		17 (68)	
	Married or cohabitating	8 (32)		7 (28)	
	Divorced	3 (12)		1 (4)	
**Device use, n (%)**					
	Use a smartphone	20 (80)		22 (88)	
	Use a PC	20 (80)		18 (72)	
	Use a tablet	10 (62.5)		12 (48)	
**Confidence in using new technology, mean (SD)**					
	0: not confident and 10: very confident	7.8 (2.5)		7.7 (2.5)	

^a^VIC: Virtual Multimedia Interactive Informed Consent.

^b^GED: General Educational Development.

### Study Procedure

Individuals who were interested in and eligible for the VIC study consented to participate in the study and completed an initial demographic survey. The participants were then randomized and scheduled for their GenEx 2.0 study visit, which usually occurred within 1 to 2 weeks of enrollment in the VIC study. If assigned to paper consent for the GenEx 2.0 trial, the individual received a copy of the paper consent in the mail to review before the study visit. Because VIC is a web-based application that provides access to the consent before, during, and after the study visit, the individuals who were assigned to the VIC received an email with a link to the remote web-based version of the VIC consent session. This remote “review” link allowed VIC participants to preview what would be seen on the iPad during the study visit, but did not give access to the final signature needed to complete the consent process. Only VIC-assigned participants had access to the application itself during this time. If VIC participants had questions or concerns, they could leave comments in the “Notes” section of each slide, which would be addressed during the study visit. Remote sessions were separately tracked in the system to differentiate between the remote and study visit sessions. 10 of 25 participants accessed the remote session before their study visit.

During the GenEx 2.0 study visit, individuals completed the consent process according to the arm to which they were assigned. Individuals assigned to the control arm (traditional paper consent) completed the consent with a study coordinator who explained each section of the consent by reading it out loud and answering any questions during the process. The individual then signed the consent form if he or she wanted to participate in GenEx 2.0. Participants were not blinded, as it was not possible for the purposes of this study, and the study coordinator was aware of which group each participant belonged to as they oversaw both groups separately. The participants were also aware that the VIC was the comparator of interest for this trial.

Individuals assigned to the intervention arm (VIC tablet-based consent) completed the visit with a study coordinator who provided the individual with an iPad along with disposable headphones so that the individual could listen to the audio instructions comfortably. The individual would then go through the consent process alone and sign the consent on the iPad at the end if they were interested in participating in the GenEx 2.0 study. The format of the VIC process on the iPad allowed for the presentation of content to be displayed 1 section at a time with a “Continue” and “Back” button that participants could press to move forward or backward. For text-only sections, no more than approximately 90 words at a time were displayed, with an average of 76.6 words per slide overall. Some sections were transformed into interactive multimedia components, including animated videos that explained study procedures (videos specific to the GenEx 2.0 trial included demonstrations of blood draw and sputum collection) as well as videos specific to privacy and withdrawal information. In addition, these multimedia components also allowed interactivity with a simple menu that could pause, play, rewind, mute, and enable closed captioning if needed. Some sections were followed by interactive quizzes that emphasized key information to enhance participant comprehension, which would give automatic feedback to the participant on the answer and allow them to go back to the key section and revisit the material, or move forward, regardless of their answer. This method did not inhibit the participant, but rather encouraged the active retention of the material. The participants were able to either continue through each section (introduction, study procedures, risks, privacy, withdrawal, and so on) in order, or access a menu that allowed the participant to view any section of the consent in the order they wished. The VIC required that the participant views each section of the study’s IC before allowing access to the signature portion of the tool. Once the IC was signed, the tool then ended and emailed a copy of the consent with the signature to the participant’s enrolled email. Once the consents were collected, participants from both groups started the GenEx 2.0 study procedures.

After completing the GenEx 2.0 study session, the participants who enrolled in the VIC study from each arm were provided with a paper exit survey that asked questions about the feasibility of their respective IC process. After completing the survey, an incentive of US $60 was provided to thank them for their time in completing both the VIC trial and the GenEx 2.0 parent study. This incentive was the same for those who did not enroll in the VIC trial and completed only the GenEx 2.0 parent study.

### Survey Assessment of Participant Comprehension and Satisfaction

The exit survey provided at the end of the GenEx 2.0 study procedures was designed to assess comprehension and satisfaction of the participants from each IC method (Multimedia Appendix 2). It included 13 comprehension questions that were structured as multiple-choice questions and were based on the validated Health Information Technology Usability Evaluation Scale and quality of IC surveys [26,28,29]. These comprehension questions measured six basic components of the IC—(1) why we asked individuals to participate in the GenEx 2.0 study, (2) the risks and benefits associated with the study, (3) their rights as participants, (4) whom to contact with questions or problems regarding the study, (5) study-specific procedures, and (6) coverage for potential study-related injuries.

The remaining 12 survey questions were administered using a 7-point Likert scale. Of these 12 questions, 4 questions assessed the participants’ self-assessed understanding of the IC general concepts, with scores ranging from 1 (I did not understand this at all) to 7 (I understood this very well); 3 questions assessed satisfaction with the IC process, with scores ranging from 1 (Very dissatisfied) to 7 (Very satisfied); 1 question assessed the perceived length of the IC process, with scores ranging from 1 (Very long) to 7 (Very short); 1 question assessed the perceived difficulty in completing the IC process, with scores ranging from 1 (Very difficult) to 7 (Very easy); 2 questions assessed the likelihood of participating in future clinical trials, with scores ranging from 1 (Very unlikely) to 7 (Very likely); and 1 question assessed the importance of the IC process in the participant’s decision to ultimately participate in the trial, with scores ranging from 1 (Not important at all) to 7 (Very important). Participants were also asked to provide an estimate of their perceived time (in minutes) to complete the IC process.

### Study Outcomes

We measured six outcomes to assess the feasibility of VIC compared with the standard paper consent in a real-world clinical research study, which were recorded through a paper survey after completing the IC process. The study outcomes included each participant’s (1) comprehension of the GenEx 2.0 study IC content, measured through a 13-question comprehension quiz; (2) satisfaction with the IC process, ranked on a 7-point Likert scale; (3) perceived time required to complete the IC process; (4) perceived ease of the IC process; (5) perceived likelihood of participating in future clinical trials; and (6) perception of the importance of IC in the decision to participate in clinical trials.

### Data Analysis Plan

Data were analyzed according to intention-to-treat analysis (ie, all participants were analyzed as randomized). Baseline data are reported as mean and SD for continuous data and as counts and percentages of total for discrete data. The 13 comprehension questions were summarized as mean (SD) and proportions (SE). Differences in the mean values of correct answers between VIC and paper consent were presented with 95% CIs. The proportion of correct answers for each of the 13 individual questions was analyzed using risk ratios (VIC relative to paper) with 95% CIs. Likert scale data for each treatment group were summarized as mean (SD), as well as the perceived time to complete the IC process, and differences in the distribution between VIC and paper were tested using the Cochran–Mantel–Haenszel statistic. One participant in each treatment group reported 120 minutes as the perceived time, which were likely outliers and were removed from the mean calculations. Analyses were performed using SAS (version 9.4; SAS Institute).

### Ethics and Security

The VIC trial protocol was approved by the Yale University Institutional Review Board. The research team included a chair of the institutional review board, who assisted with human rights perspectives. We anticipated potential risks with study participants consenting to a study via a digital medium and took multiple precautions to ensure comprehension of the digital IC for the GenEx 2.0 trial. The tool was tested with multiple users and subject matter experts before the study to determine its usability. The research coordinator had the ability to review the quiz results of the participant, as well as the time taken to read each section of the consent. The research coordinator evaluated each participant’s quiz scores and the time spent going through the consent to determine if the participant was properly informed. If the research coordinator did not feel the participant understood the nature of the study, the coordinator had the ability to withdraw the consent.

The VIC tool was hosted on a secure server located on the Yale University network and only accessible to those with explicit administrator access via the Yale University network through Yale’s Central Authentication System. Databases storing participant data were also maintained on the Yale University network and hosted on separate servers from the tool itself to increase security measures. The databases were backed up and stored securely in a separate location on the Yale University network with access granted only to the system administrator and principal investigator. As with any digital application, we planned and anticipated common issues such as bugs, and although no bugs occurred during the trial itself, the developer was available to maintain the system and ensure stability of the application.

## Results

### Eligibility

**Figure 2:**
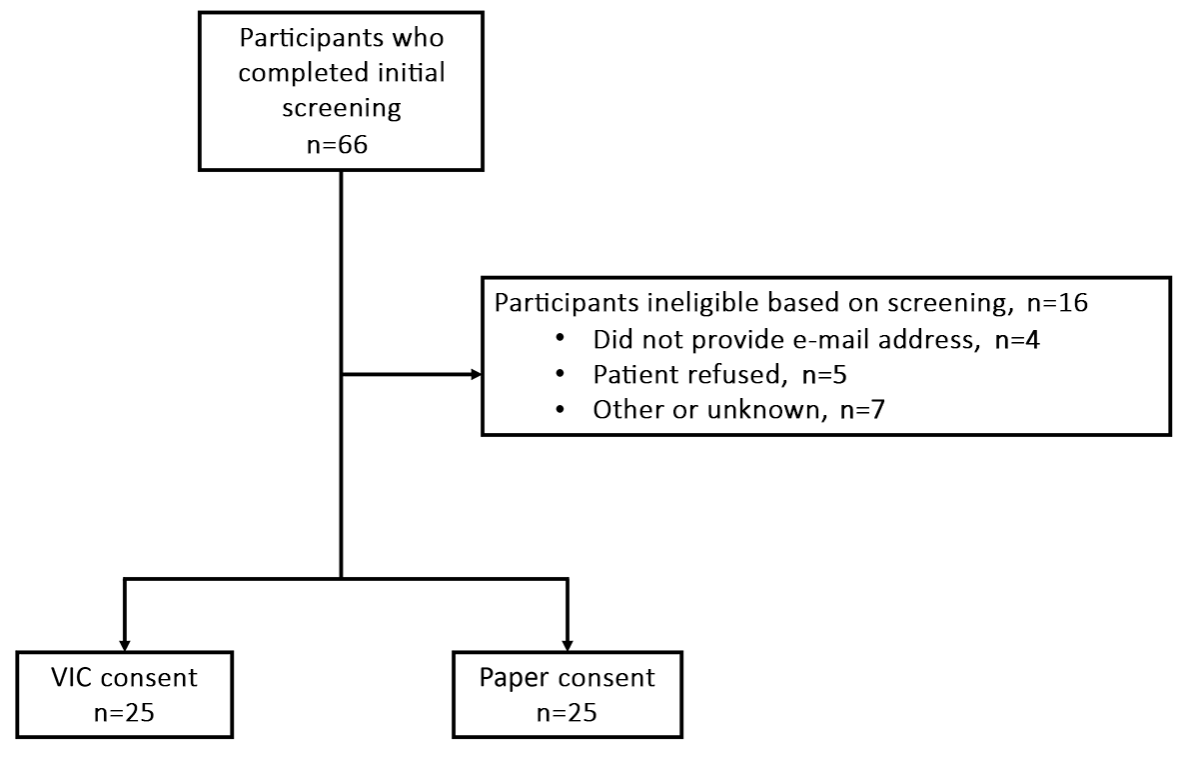
Flow diagram of participant enrollment in the Virtual Multimedia Interactive Informed Consent trial CONSORT (Consolidated Standards of Reporting Trials) diagram. VIC: Virtual Multimedia Interactive Informed Consent.

A total of 91 individuals were approached for the study, of whom 25 were ineligible because they had already participated in GenEx 2.0. The remaining 66 individuals completed the initial screening questionnaire, and of them, 16 participants were deemed ineligible because they either did not provide an email address (n=4), refused to participate (n=5), or other unknown reasons (n=7). A total of 50 individuals were ultimately enrolled in the VIC trial, and 25 were randomized to the VIC intervention arm and 25 to the paper IC control arm (Figure 2).

### Demographics

The mean age of the participants enrolled was higher in the VIC arm (47.0, SD 15.3 years) than in the paper arm (37.7, SD 14.7 years); with a mean difference 9.4 (95% CI 0.9-17.9). Overall, other demographic characteristics, such as race, ethnicity, household income, and relationship status, were comparable between both arms (Table 1). Regarding employment, 52% (13/25) of the VIC arm participants and 60% (15/25) of the paper consent group participants were employed at least part-time; education level was similar across both arms (Table 1). Most participants in both arms reported using or having access to a smartphone (20/25, 80% VIC arm, 22/25, 88% paper arm), less than half reported using or having access to tablets (10/25, 40% VIC arm vs 12/25, 48% paper arm), and most used or had access to PCs (20/25, 80% VIC arm vs 18/25, 72% paper arm). When asked about confidence in using new technology, participants in both arms reported similar levels of confidence on an 11-point Likert scale with 0 being “Not confident at all” and 10 being “Very confident” (mean 7.8 VIC vs 7.7 paper).

### Survey Results

Overall, participant comprehension was high in both the IC process groups. For the comprehension outcome (Table 2), VIC participants scored a mean of 11 correct answers of 13 compared with the paper IC group mean of 10.6 correct answers; the mean difference in the number of correct answers between VIC and paper group was 0.4 (95% CI −0.5 to 1.3). For each of the 13 individual comprehension survey questions, the proportion of correct responses was generally comparable for VIC and paper group with risk ratios not different from one (Table 2). However, VIC participants appeared to have better knowledge about the use of their personal health information (PHI) and study withdrawal (risk ratios>1.20), which were sections in the tablet-based tool formatted uniquely as animated videos rather than plain text.

**Table 2:** Proportion of correct responses on the Participant Comprehension Survey by assigned study arm (N=50).

Number	Question	Participants with correct answers, n (%)		VIC^a^ vs paper, risk ratio (95% CI)
		VIC arm (n=25)	Paper arm (n=25)	
1	Why did we ask you to participate in this study?	18 (72)	20 (80)	0.90 (0.66-1.23)
2	Which of the following are benefits of this study?	18 (72)	17 (68)	1.06 (0.74-1.52)
3	Which of the following procedures is part of the study?	25 (100)	24 (96)	1.04 (0.96-1.13)
4	Which of the following is true if you choose to participate?	23 (92)	24 (96)	0.96 (0.83-1.10)
5	Which of the following is an expected risk from participating in the study?	22 (88)	20 (80)	1.10 (0.86-1.40)
6	While you are in this research study, what will happen to your personal health information?	24 (96)	19 (76)	1.26 (1.00-1.60)
7	Which of the following statement is true about your participation in the study?	20 (80)	18 (72)	1.11 (0.81-1.52)
8	How can you withdraw from this study?	24 (96)	20 (80)	1.20 (0.97-1.48)
9	Who can you call if you have questions about your rights as a participant in this study?	18 (72)	16 (64)	1.13 (0.77-1.65)
10	Your driver’s license number will be collected for purposes of this study	25 (100)	25 (100)	1.00 (1.00-1.00)
11	To participate in this study, you need to provide blood sample	24 (96)	25 (100)	0.96 (0.89-1.04)
12	If you are injured while participating in this study, you or your insurance carrier will be expected to pay the costs of this treatment	9 (36)	12 (48)	1.23 (0.76-1.99)
13	If you participate in the procedures for this study, you will be paid for your time	25 (100)	25 (100)	1.00 (1.00-1.00)

^a^VIC: Virtual Multimedia Interactive Informed Consent.

**Figure 3:**
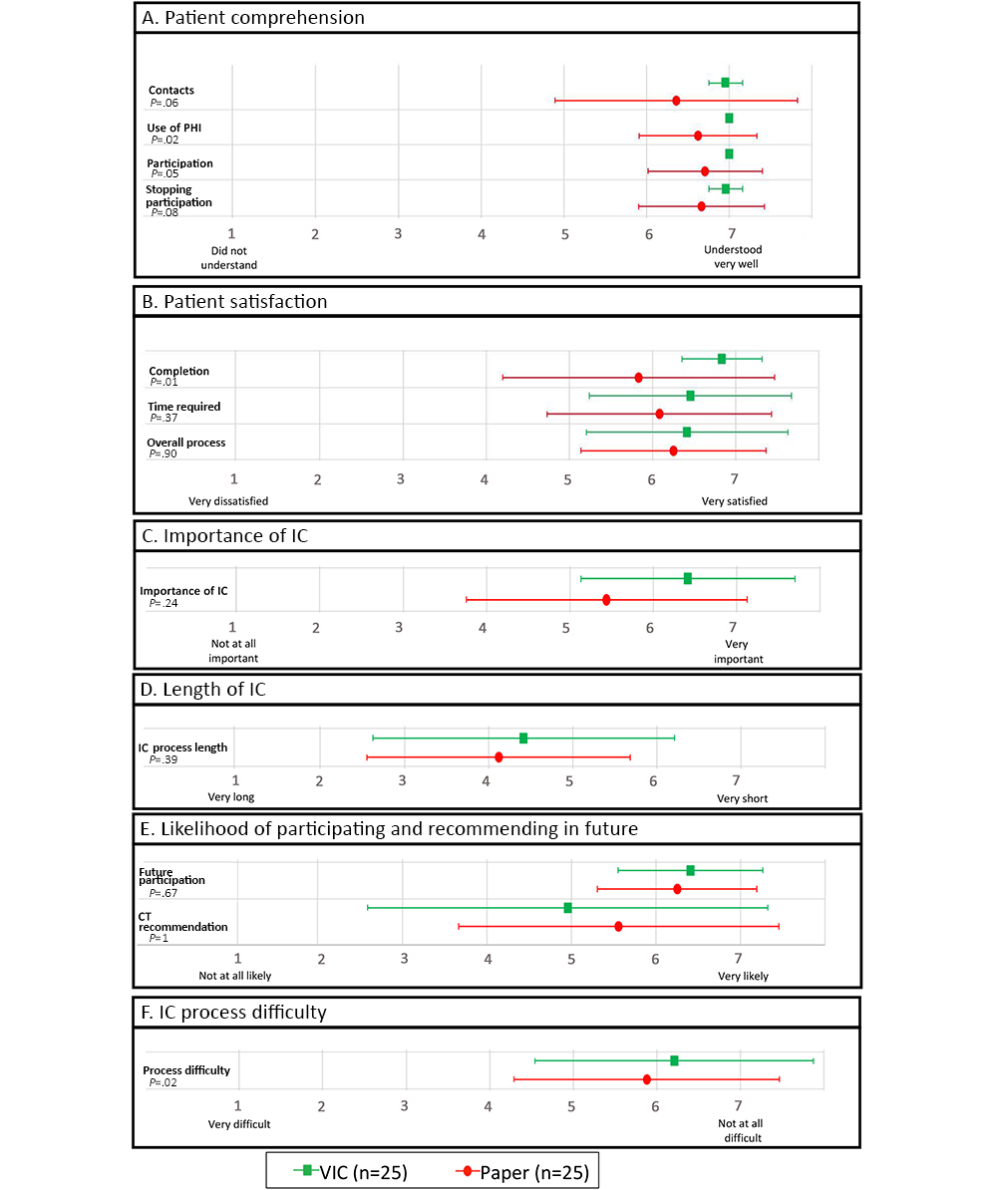
Perceived participant comprehension and satisfaction by the type of informed consent (IC) based on a 7-point Likert scale of levels of comprehension, satisfaction, perceived importance, perceived length, participation in clinical trials, and IC process difficulty. Mean (SD) values are plotted in the figure along with Cochran–Mantel–Haenszel test *P* values. CT: clinical trial; IC: informed consent; PHI: personal health information; VIC: Virtual Multimedia Interactive Informed Consent.

The participants were also asked questions to ascertain their level of satisfaction and perception with the 2 consent processes (Figure 3). Overall, mean levels of satisfaction and perception on a 7-point Likert scale were higher with the VIC tool for every category except for the “Clinical Trial Recommendation” category. When participants were asked how well they understood whom to contact with any questions or concerns related to the research study, VIC participants had a higher mean score (7.0) than the paper consent participants (6.4) (*P*=.05).

Furthermore, on average, VIC participants had a slightly better understanding of who in the study had access to their PHI and what would happen if they chose to participate in the study (mean scores of 7 for both), although paper consent participants scored a mean of 6.6 and 6.7, respectively (*P*=.02 and *P*=.045, respectively). When asked about their level of understanding on how to withdraw from the study, VIC participants had a mean score of 7.0 versus 6.7 for paper consent participants. Furthermore, VIC participants reported a lower mean perceived time to complete the consent process, 12.9 (SD 7.6) minutes for VIC participants versus 16.6 (SD 9.7) minutes for paper consent participants; mean difference of −3.7 (95% CI −9.0 to 1.5) minutes. In terms of satisfaction with completion time, VIC participants had a higher mean score than paper consent participants (6.8 vs 5.8; *P*=.01). Regarding overall process difficulty, VIC participants scored the process as less difficult, with a mean score of 6.3 compared with paper consent participants with a mean score of 5.9 (*P*=.02).

Regarding satisfaction with the overall consent process (Figure 3), VIC participants reported an average satisfaction level of 6.4 (7 being “Very satisfied”), whereas paper consent participants had an average satisfaction level of 6.3. More specifically, VIC participants reported an average satisfaction level of 6.8 for their ability to complete the consent process on their own without any help from research staff versus 5.8 for paper consent participants. VIC participants also scored a slightly higher average of 6.4 for their satisfaction with the time required to complete the IC process, whereas paper consent participants scored an average of 6.1. Finally, participants in the VIC group found the process of completing the IC process to be easier than participants in the paper group (6.4 VIC vs 5.4 paper).

The VIC participants were also more likely to recommend that research studies in the future use their method of IC (ie, electronic) than participants from the paper consent group (6.4 VIC vs 5.9 paper). Both IC groups reported that they were very likely to participate in the future clinical trials (6.4 VIC vs 6.3 paper) and that the IC process was relatively important in their decision to participate in the clinical research study (5.0 VIC vs 5.7 paper).

## Discussion

### Principal Findings

The study was conducted to test the feasibility of the VIC digital consent tool that enhances the IC process with interactive content on tablets. We built a VIC tool with a reusable infrastructure that allows for integration into the IC process for future research studies and may improve the clinical workflow through a more efficient IC process. The VIC tool can assess participant comprehension through automated quizzes and self-tests, emphasize topics using multimedia, and allow individuals to view demos and presentations. The participants can also listen to comments and explanations, get customized information, click on links to drill down for more information, ask questions, receive answers, and rewind and replay audio and visual components as needed. The VIC tool also provides a retrievable electronic record of the IC document, including the electronic signature, which can be integrated into modern electronic health records. Although not performed for the purposes of this study, VIC can also be performed remotely and securely with the electronic signature feature, and on a PC or smartphone if needed.

### Significance

These innovative, dynamic features enhanced the overall participant experience in the ongoing research study, GenEx 2.0, compared with the standard paper IC process. In our study, the VIC trial participants reported significantly better understanding of the use of their PHI and participation in the study and greater satisfaction in completing the IC process because of the shorter duration. Previous systematic reviews have suggested that enhanced consent forms and extended discussions are most effective in improving participant understanding, and our findings confirm this claim [3,28-32]. This study provides additional evidence and reinforces the findings of previous studies implying that using digital health may enhance participant comprehension.

Systematic reviews have also suggested that the integration of audiovisual elements into the IC process can improve participant recall with no adverse impact on satisfaction or anxiety [33], and we similarly found that the use of an iPad for VIC did not negatively affect participants’ willingness to participate or their satisfaction with the process. For the purposes of this study, the VIC tool used 5 videos (with closed caption options) in place of text. These videos were moderately dispersed throughout the tool, and the VIC ultimately contained less text than the paper version of the consent, which may have aided in reducing consent fatigue. It is important to note that VIC is not simply an electronic version of the IC process, but contains interactive multimedia components that visually demonstrate study procedures and certain sections of the consent document not normally seen in the traditional methods. Some studies referring to electronic consent processes may simply be a plain text version of the paper IC consent that offers text-to-speech as an audio feature and thus differ from VIC in this way.

A particular strength of our study was the option of having the participants provide consent to participate in an actual ongoing parent study in which they were enrolled prospectively, rather than asking them to imagine that they would take part in a hypothetical study. The real-world aspect of the study ensured that we were actively collecting data instead of relying on retrospective analyses, which have inherent limitations [26,27].

One comprehension question that provided indication of poor content delivery in both arms was the question of whether participants believed that they or their insurance carriers would be expected to pay for the costs of the treatment if they were injured while participating in the study. In the VIC arm, 64% (16/25) of the participants answered incorrectly compared with 52% (13/25) of those who answered incorrectly in the paper arm. In contrast, the other 12 questions indicated moderate to high comprehension of the content in both arms. This may be a reflection of the difficulty conveying language typical to the consent form regarding this specific section, because our results indicate it is atypical to the positive trend.

### Limitations

There are some limitations with regard to the nature of this study, which may have affected certain findings. One limitation of this study was that we did not have masked research staff administering the survey to participants after the IC process. The study coordinator for GenEx 2.0 was the same person who administered the paper-based and VIC IC processes and collected study surveys, which limits our control over observer bias. Another limitation was that we did not directly and independently measure the time to complete the IC and instead relied on the participant-reported perceived time to complete the IC. We also limited participation in this study to individuals who spoke English, had an email address, and were willing to use the iPad. Although most participants were confident in using technology, we believe that these conditions could have potentially limited the generalizability of the findings because 6% (4/66) of the screened individuals did not provide an email address. Although the GenEx 2.0 study topic difficulty level could be described as moderate with very few study procedures involved, VIC may perform significantly better with more complicated topics with the use of multimedia and dynamic features to reinforce information, such as the “teach-back” quiz feature. In addition, our sample size of 50 participants could have affected the ability to detect differences between VIC consent participants and paper consent participants, which we plan to address in future research regarding the tool.

Potential future research topics that would be important to consider and explore with the VIC tool would be expanding future research using VIC to include participants of other languages other than English, as well as population with various hearing and vision impairments. The integrated text-to-speech and other audiovisual components of VIC are innovative language integration tools that would make switching to consent in other languages a very feasible task. Moreover, the tablet-based methods of VIC also offer various accessibility tools that can be considered to reach a more inclusive target population, and with VIC’s primary feature prioritizing a customized level of information, we feel that there is great potential for future topics to maximize its usability. Although cost analysis was not performed for this study, we believe that in future studies, the cost of creating a dynamic, digital IC may lessen as the tools become scalable and feasible to others.

Our team plans to disseminate the VIC tool in collaboration with other institutes to facilitate the adaptation of digital consent platforms with the goal of creating a scalable, dynamic, and effective IC process.

### Conclusions

This study found that the VIC tool is feasible when integrated into a real-world research study, and the use of multimedia and other interactive features via a tablet-based IC process led to greater satisfaction in delivering important content compared with the standard paper process. VIC participants reported a lower perceived time to complete the IC process and higher comprehension, as well as higher overall satisfaction compared with the participants in the control group. Our preliminary findings suggest that compared with the standard paper consent process, dynamic digital IC processes can enhance comprehension and satisfaction and transform the consent process for human-based research studies.

## Appendix

Multimedia Appendix 1 Virtual Multimedia Interactive Informed Consent demo video.

Multimedia Appendix 2 Virtual Multimedia Interactive Informed Consent trial survey.

Multimedia Appendix 3 CONSORT-eHEALTH checklist (V 1.6.1).

## Abbreviations

IC: informed consent
PHI: personal health information
VIC: Virtual Multimedia Interactive Informed Consent
WCC: Winchester Chest Clinic
