# Supplementary material for: Comparing a Multimedia Digital Informed Consent Tool With Traditional Paper-Based Methods: Randomized Controlled Trial
Source: JMIR Form Res. 2021 Oct 19;5(10):e20458. doi: 10.2196/20458 (PMC8564662; doi:10.2196/20458)
Supplement: Multimedia Appendix 2 [file formative_v5i10e20458_app2.pdf]

VIC Study Screening Date:____/____/______ Study ID: ____________________

Yale NewHaven Health

YALE-NEW HAVEN HOSPITAL

Please estimate how long you will spend completing the forms and informed consent for this VIC research study: ____________ minutes

Please read each statement carefully, and check the SINGLE BEST RESPONSE.

1. Why did we ask you to participate in this study?
   - ❑ To gather samples for lung diseases research.
   - ❑ To test a new asthma medicine.
   - ❑ To provide samples for cancer research.
   - ❑ All of the above.

2. Which of the following are benefits of this study?
   - ❑ It will make me receive better care at the clinic.
   - ❑ It may help in finding new discoveries about lung diseases.
   - ❑ There are no benefits.
   - ❑ All of the above.

3. Which of the following procedures is part of the study?
   - ❑ Blood draw.
   - ❑ Lung function testing.
   - ❑ Sputum collection.
   - ❑ All of the above.

4. Which of the following is true if you choose to participate?
   - ❑ A sample of your blood will be collected and stored for research.
   - ❑ You cannot quit without our approval.
   - ❑ You will not be asked to participate in lung function test.
   - ❑ You will be get large payment if we discover new medicine.
5. Which of the following is an expected risk from participating in the study?
   - ❑ You may feel faint or dizzy during blood collection
   - ❑ You may start to wheeze or cough following these tests.
   - ❑ The test will be monitored closely and it will be stopped if your breathing or lung function gets worse.
   - ❑ All of the above.

6. While you are in this research study, what will happen to your personal health information?
   - ❑ It will be shared by the researchers with anyone who asks for it.
   - ❑ It will not be collected or used by researchers in this research study.
   - ❑ It will be used by my personal physician ONLY.
   - ❑ It will be shared with physicians, nurses and staff working on this research study ONLY.

7. In which of the following circumstances can you stop participating in the study?
   - ❑ Your participation in this study is voluntary, you can stop being in the study at any time.
   - ❑ If you chose to stop being in the study, no one will treat you differently and you will not be penalized.
   - ❑ If you chose to stop being in the study, the data obtained from the specimens up to the point of your withdrawal will continue to be used.
   - ❑ All of the above.

8. How can you withdraw from this study?
   - ❑ Call my healthcare provider
   - ❑ Call my insurance company
   - ❑ Call Yale University
   - ❑ Call the head of the study, Dr. Geoffrey Chupp.

9. Who can you call if you have questions about your rights as a participant in this study?
   - ❑ You may contact the head of the study, Geoffrey Chupp.
   - ❑ You may contact the Human Investigation Committee.
   - ❑ You can contact the Yale Privacy Officer.
   - ❑ All of the above.

10. Regarding your participation in the study, please check **TRUE** or **FALSE** for the following statements.

	**True**	**False**
Your driver's license number will be collected for purposes of this study	☐	☐
To participate in this study, you need to provide blood sample	☐	☐
If you are injured while participating in this study, You or your insurance carrier will be expected to pay the costs of this treatment	☐	☐
If you participate in the procedures for this study, you will be paid for your time	☐	☐

On a scale of 1 to 7, How well do you understand the following statements?
DIDN'T UNDERSTAND THE ITEM AT ALL, CHECK 1. UNDERSTOOD IT VERY WELL, CHECK 7.

	I did not understand this at all			⟷			I understood this very well
Whom you should contact if you have questions or concerns related to the research study	1	2	3	4	5	6	7
Who will have access to your protected health information	1	2	3	4	5	6	7
What will happen if you choose to participate in this research study	1	2	3	4	5	6	7
How you can stop participating in the study	1	2	3	4	5	6	7

On a scale of 1 to 7, How satisfied were you with the following statements?
VERY DISSATISFIED, PLEASE CHECK 1. VERY SATISFIED, PLEASE CHECK 7.

	Very dissatisfied			⟷				Very satisfied
Your ability to complete the informed consent process for this research study on your own, without any staff	1	2		3	4	5	6	7
The time required to complete the informed consent process.	1	2		3	4	5	6	7
The overall informed consent process	1	2		3	4	5	6	7

On a scale of 1 to 7, How long do you think the informed consent process was?
A VERY LONG PROCESS, PLEASE CHECK 1. A VERY SHORT PROCESS, PLEASE CHECK 7.

	Very long ⟷ Very short						
Thinking about the entire "informed consent process" would you say it was	1	2	3	4	5	6	7

On a scale of 1 to 7, How difficult did you feel the informed consent process was?
VERY DIFFICULT, PLEASE CHECK 1. VERY EASY, PLEASE CHECK 7.

	Very difficult ⟷ Very easy						
The process of completing the informed consent process was	1	2	3	4	5	6	7

On a scale of 1 to 7, How likely are you to participate in a future clinical trial.
NOT LIKELY AT ALL, PLEASE CHECK 1. VERY LIKELY, PLEASE CHECK 7.

	Very unlikely ⟷ Very likely						
How likely are you to join another research study?	1	2	3	4	5	6	7
Would you recommend that research studies in the future use the same type of informed consent format used for THIS study?	1	2	3	4	5	6	7

On a scale of 1 to 7, How important was the informed consent process in your decision to participate in this clinical research study?
NOT IMPORTANT AT ALL, PLEASE CHECK 1. VERY IMPORTANT, PLEASE CHECK 7.

	Not important at all ⟷ Very important						
Please rate how important the informed consent process was in your decision to participate in the research study	1	2	3	4	5	6	7
